# The effect of modality onset asynchrony and processing time on the recognition of text-supplemented speech

**DOI:** 10.1121/10.0017215

**Published:** 2023-02-15

**Authors:** Kimberly G. Smith, Daniel Fogerty

**Affiliations:** 1 Department of Speech Pathology and Audiology, University of South Alabama, 5721 USA Drive North, Mobile, Alabama 36688, USA; 2 Department of Speech and Hearing Science, University of Illinois Urbana-Champaign, 901 South Sixth Street, Champaign, Illinois 61820, USA kimberlysmith@southalabama.edu, dfogerty@illinois.edu

## Abstract

This study examined the effect of modality onset asynchrony and response processing time for the recognition of text-supplemented speech. Speech and text were periodically interrupted by noise or black bars, respectively, to preserve 50% of the sentence and presented in unimodal and multimodal conditions. Sentence recognition and response errors were assessed for responses made simultaneous with the stimulus or after its presentation. Increased processing time allowed for the cognitive repair of initial response errors in working memory. Text-supplemented speech was best recognized with minimal temporal asynchrony. Overall, text supplementation facilitated the recognition of degraded speech when provided sufficient processing time.

## 1. Introduction

Communicating in everyday environments often entails listening to speech that is degraded, such as due to background noise. Such perceptually demanding conditions require individuals to rely on partial information that can be glimpsed from the degraded signal. Although useful glimpses of speech information can be exploited (Cooke, 2006), extremely degraded conditions, or the presence of additional factors such as hearing loss, can limit the utility of highly sparse glimpses of speech. One strategy to facilitate speech recognition in such degraded contexts is to supplement speech with a text display (Zhong *et al.*, 2022). Text supplementation of degraded speech has been shown to improve speech recognition [e.g., Krull and Humes (2016), Smith and Fogerty (2015, 2016), and Zekveld *et al.* (2008)] and enhance memory and later recall of the degraded message (Payne *et al.*, 2022). Indeed, such multimodal presentations of speech and text provide advantages to sentence recognition over either degraded modality alone (Fogerty *et al.*, 2020; Fogerty *et al.*, 2022; Smith and Fogerty, 2015, 2016). However, deriving text captions from noisy speech results in errors in the text transcription (Krull *et al.*, 2013) which can significantly impair recognition and memory for the multimodal signal (Crandell *et al.*, 2022). Thus, it is important to detail how listeners benefit from text supplementation when both speech and text modalities contain partial information. This study investigates this real-world problem of partial information by degrading speech and text using periodic interruptions.

Several stimulus factors influence the effectiveness of processing text-supplemented speech. These factors include how much of the speech or text is preserved [e.g., Fogerty *et al.* (2022)], the distribution of glimpses across modalities (Fogerty *et al.*, 2022), and the relative timing of the two modalities [e.g., Zekveld *et al.* (2008)]. The current study examines the effect of the relative timing between modalities in more detail, across a range of modality onset asynchronies. Thus, this study examines the time at which listeners are most receptive to the presence of degraded text supplementation.

In addition to these stimulus level factors, procedural factors related to the timing of the behavioral response also affects recognition. That is, behaviorally shadowing the stimulus through a simultaneous response provides an online measure of speech recognition as the stimulus unfolds [e.g., Cherry (1953)]. In contrast, most speech recognition paradigms cue listeners to respond after the stimulus presentation. This listen-and-respond procedure provides a measure of speech recognition that includes the potential, following the stimulus presentation, for cognitive repair of misperceived words [e.g., Winn and Teece (2021)]. Importantly, such a delay in responding may also allow additional processing time for the integration or comparison of information between speech and text, thereby enhancing the benefit of text supplementation. This additional time may be particularly important for modality integration when speech and text do not provide correlated perceptual cues and are only associated at later linguistic processing stages. Consistent with the potential effects of cognitive repair and modality integration, results have confirmed that delaying the behavioral response results in better recognition compared to a simultaneous response (Smith and Fogerty, 2015, 2016). A further question regards the amount of speech information that remains in memory following the initial response [e.g., Payne *et al.* (2022)]. By providing an opportunity for a second recall response at a standard time interval following the stimulus, this study directly examines the effect of responding while listening, as well as measures the perceptual benefit obtained by providing additional processing time for post-stimulus revisions of the sentence representation in memory. Alternatively, the additional delay imposed for a second response could result in a decay in memory for the sentence, particularly if it is poorly represented, such as due to stimulus degradation [e.g., Rabbitt (1968) and Surprenant (1999)].

To complement information obtained from average speech recognition scores, error analyses have been conducted to provide a more detailed account of speech recognition across listening conditions and listener groups [e.g., Fogerty *et al.* (2020), Smith and Fogerty (2017, 2021), and Zinszer *et al.* (2019)]. Smith and Fogerty (2017, 2021) coded speech recognition errors of omission, substitution, and addition for phonemes and words. Their studies have shown that younger normal hearing adults are more likely to substitute or omit entire words, depending on the level of degradation, with fewer errors associated with phoneme-level confusions. Older adults were even more biased in this process for word-level errors. These findings are consistent with an interactive system in which higher level linguistic processes compensate for degraded perceptual information [e.g., the ELU model; Rönnberg *et al.* (2013)], potentially during a postprocessing stage of cognitively repairing the original heard utterance (Winn and Teece, 2021). Even non-native listeners with incomplete language knowledge demonstrate greater content-level word errors (Zinszer *et al.*, 2019). Examining these response errors across different timings of the response can potentially capture how additional processing time allows for the recruitment of top-down linguistic processes that may resolve perceptual confusions. In this way, such an analysis may reveal how top-down processing is used to repair initial, online predictions of the sentence when the listener is afforded later processing time.

As detailed above, several factors can influence multimodal sentence recognition. Processing time is one of these main factors that may influence the success of comparing and combining information from the two modalities, as well as providing time for later cognitive repair of initial misperceptions. In the current study we examined timing in three ways. First, the relative timing between the speech and text (*modality onset asynchrony*) determines the duration at which speech and text are available for processing separately and together. Consistent with earlier findings (Zekveld *et al.*, 2008), we expected the best performance when the two modalities are temporally aligned at the onset. Second, we compared responses that were simultaneous or delayed (*response timing*) relative to the speech stimulus. Third, we compared these initial, first responses to responses made during a later, second recall interval (*recall interval*). We hypothesized that additional processing time, provided either by a delayed response or a second recall interval, would enhance recognition compared to the simultaneous or initial recall of the sentence, respectively. Last, we also expected this additional processing time to allow for resolving initial perceptual confusions, resulting in fewer phoneme-level confusions and shifting word-level errors from omissions to substitutions at the second recall interval. Therefore, this study examined the effect of modality onset asynchrony, response timing, and recall interval on the recognition accuracy and perceptual errors of degraded text-supplemented speech.

## 2. Methods

### 2.1 Participants

Twenty young adults (*M* = 20.45, *SD* = 1.43 years of age; 11 females, 9 males) who were native speakers of American English participated in data collection for this study. All participants had normal hearing based on a pure-tone hearing screening at 20 dB HL at octave frequencies from 500 to 8000 Hz (ANSI, 2004). Participants also had normal vision based on screening using the Good-Lite near-vision standard reading test card [Near Point Reading Card “Clearing weather” (Good-Lite, 2023)], which tests reading vision acuity using text examples from newspapers and books at a distance of 16 inches. All participants reported an unremarkable history of any speech, language, reading, or cognitive difficulties. No participants were excluded based on the self-reported history, hearing, or vision screening procedures. All participants consented to participation on the basis of the protocol approved by the University of South Alabama Institutional Review Board.

### 2.2 Stimuli

The speech and text stimuli were selected from recordings of a male talker (Loizou, 2007) speaking the Institute of Electrical and Electronics Engineers (IEEE)/Harvard sentences (Egan, 1944; Rothauser *et al.*, 1969). The Harvard corpus consists of meaningful, low context sentences that each contain five key words. Speech and text stimuli were interrupted to preserve the same number of interruptions per sentence at a rate of approximately 8 Hz and a duty cycle of 50%.

Speech stimuli were interrupted by periodically alternating in time with an interrupted noise, gated with a 2-ms raised cosine on/off ramp, and presented at 3 dB above the average level of the sentence prior to interruption. The noise was spectrally shaped to match the long-term average speech spectrum of the Harvard corpus.

**Fig. 1.**
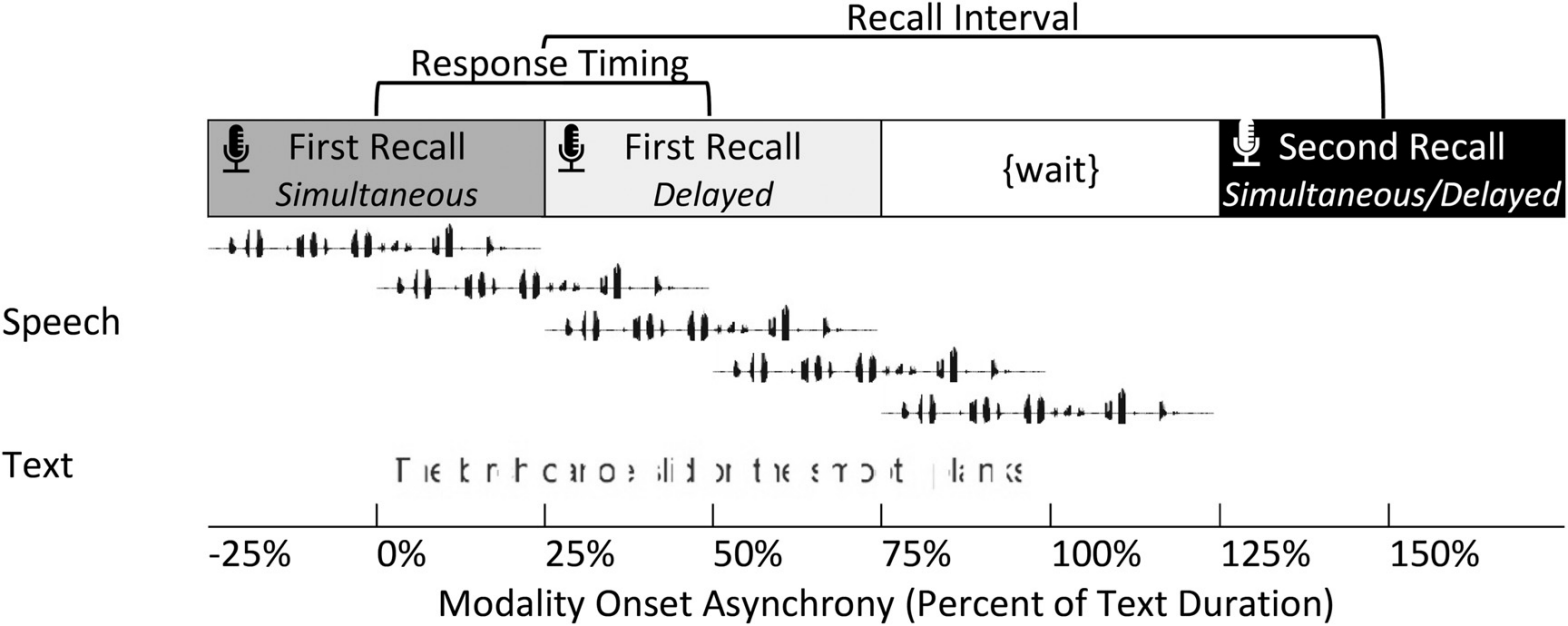
Schematic illustration of each of the five onset asynchronies for speech relative to the text presentation. The first recall interval, cued by the block as either simultaneous or delayed, is displayed for the first speech onset asynchrony (–25%). The second recall interval was the same for all conditions. The sentence in this example, “The birch canoe slid on the smooth planks,” had a speech duration of 3.1 s (text duration of 6.2 s).

Text stimuli were designed to model the interruption parameters of the speech to approximate interruption time in milliseconds in space taken in pixels [see Shafiro *et al.* (2018)]. This calibration, based on a sentence of median duration, resulted in a font size of 22.5 points on a 1600 × 200 pixel visual window with a conversion of 400 pixels to 1 s. A black bar pattern was used to mask red Arial font on a white background. The sentence was displayed on the computer monitor for twice the duration of the audio file for that specific sentence. Figure 1 displays a schematic of an example waveform and text display interrupted according to the stimulus processing.

### 2.3 Study design

To assess baseline performance, all participants first completed *unimodal testing* when only a single interrupted modality was available: text or speech. Unimodal testing was completed for the two response timings: simultaneous or delayed. For the simultaneous condition, participants spoke/read along with the speech/text as soon it began. Scoring was conducted on a second recall interval elicited after the stimulus presentation ended. For the delayed condition, participants responded once with what was heard or read once the stimulus presentation ended. Twenty sentences were presented in each condition for a total of 80 sentences (4 conditions × 20 sentences).

The *multimodal testing* used a 5 (modality onset asynchrony: −25%, 0%, 25%, 50%, 75%) × 2 (response timing: simultaneous, delayed) × 2 (recall interval: first, second) repeated measures design. Figure 1 provides a schematic to visualize the relative timing of responses and stimuli according to these three factors. The five modality onset asynchrony conditions varied the timing of the speech relative to the text based on the proportion of the text duration from −25% when half of the speech duration started before the onset of the text to 75% where half of the speech lagged behind the text. The first recall interval was cued by the block as either simultaneous or delayed response timing, and the second recall interval was the same for all conditions. Multimodal testing consisted of 10 blocks (5 modality onset asynchronies, 2 response timings; 2 recall intervals with every condition) with 20 trials per block.

### 2.4 Procedures

Listeners were seated at an individual computer terminal in a sound-attenuating booth. Speech stimuli were presented monaurally to the right ear while wearing Sennheiser HD 280 Pro headphones with 32 dB ambient noise attenuation. Five familiarization trials were presented before each experimental block. Stimuli were presented randomly within each block. Response timing and modality onset asynchrony conditions were counterbalanced across participants for multimodal testing. The test battery was completed as a single test session of about two and a half hours. Breaks were provided between conditions as needed.

Participants were instructed to respond twice for each trial. In the first recall interval, participants were directed to read the sentence aloud along with the talker (simultaneous response) or repeat the sentence aloud after the talker finished the sentence while they read along (delayed condition).^1^ In the second recall interval, participants were asked to repeat what they heard and read when visually cued on the screen after both stimuli were removed and they had completed their first recall. Participants were encouraged to guess if they were uncertain regarding the message.

### 2.5 Scoring

Participant responses were digitally recorded for later analysis, and were orthographically transcribed and error coded using the coding system from Smith and Fogerty (2017, 2021). Errors were coded based on the five key words per sentence. Seven error codes were used: three *word*, three *sound*, and one *other* error type. *Word* errors included word substitution, addition, and omission. *Sound* errors included sound substitution, addition, and omission. *Sound* errors were scored when 50% or more of the target word phonemes remained intact. Phonetic transcription was used to determine this threshold. The *other* error type was scored for any error that did not meet criteria for a word or sound error (e.g., non-word). Less than 1% of errors were coded as *other* and were therefore omitted from analyses.

One trained native English speaker transcribed and error coded all responses. To establish reliability, a second rater scored 10% of the responses. Inter-rater agreement was 95% for transcription and 91% for error coding across the two raters.

## 3. Results

### 3.1 Recognition of text-supplemented speech

Sentence recognition accuracy for each condition was entered into a 5 (modality onset asynchrony: −25%, 0%, 25%, 50%, 75%) × 2 (response timing: simultaneous, delayed) × 2 (recall interval: first, second) repeated-measures analysis of variance (ANOVA). Paired-samples t-tests with Bonferroni correction for multiple comparisons were used for *post hoc* comparisons. The results of the ANOVA are detailed in Table 1 with a brief summary of the results below.

**Table 1.** Repeated-measures ANOVA results.

	df	F	*p*	η_p_^2^
Modality onset asynchrony (−25% to 75%)	4, 76	5.93	<0.001	0.24
Response timing (simultaneous, delayed)	1, 19	129.33	<0.001	0.87
Recall interval (first, second)	1, 19	43.91	<0.001	0.70
Recall interval × response timing	1, 19	36.74	<0.001	0.66
Recall interval × modality asynchrony	4, 76	0.31	0.82	0.02
Response timing × modality asynchrony	4, 76	4.05	0.01	0.18
Recall interval × response timing × modality asynchrony	4, 76	0.86	0.47	0.04

**Fig. 2.**
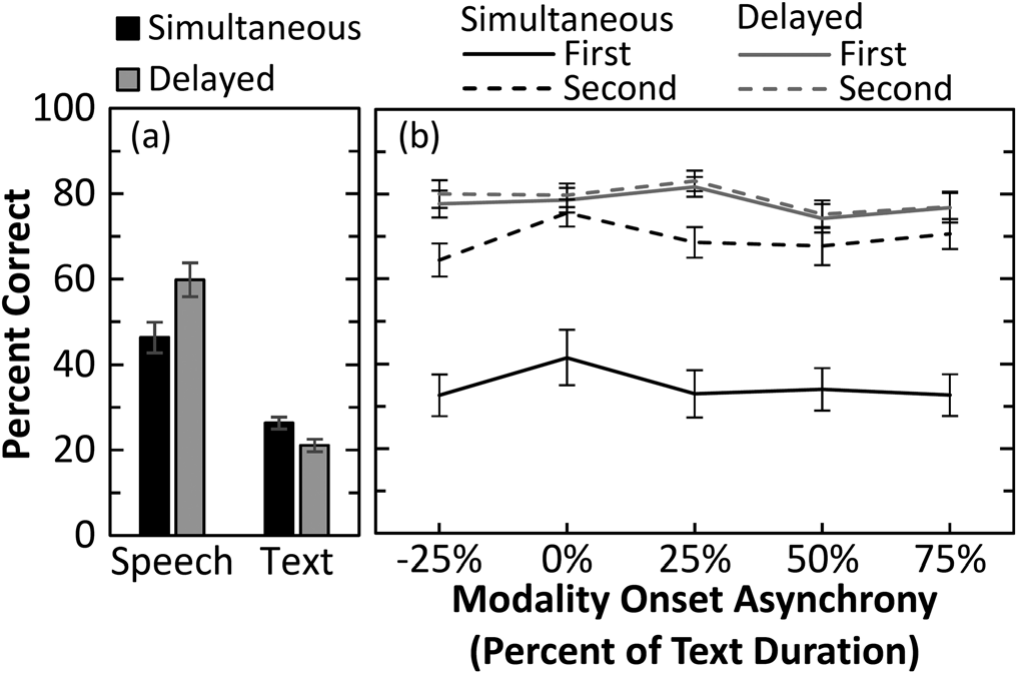
Keyword percent correct recognition for (a) unimodal (bars) and (b) multimodal (lines) tasks. Response type is indicated by the bar or line color: simultaneous (black), delayed (gray). For the multimodal conditions, response interval is coded by line style: first recall (solid), second recall (dashed), and plotted across the modality onset asynchronies as a proportion of the text duration. Error bars indicate ±1 standard error of the mean.

Significant main effects emerged for all three factors (see Table 1). Figure 2 displays keyword percent correct for each condition with the unimodal results (bars) provided as a baseline reference. Overall, sentence recognition accuracy improved with more processing time, as seen by the significant main effects, with higher scores for delayed vs simultaneous responses and for the second compared to the first response interval. The significant interaction between response timing and recall interval is observed in Fig. 2. Delaying the initial response until after the speech was presented (delayed-simultaneous) had the largest effect on the first response (solid lines) compared to the second (dashed lines; 43 vs 10%-point difference for first and second recall intervals, respectively). For modality asynchrony, an interaction occurred with response timing. Paired t-tests were conducted between modality asynchronies for each response time. For a simultaneous first response, recognition at 0% onset asynchrony was similar or better (compared to –25% and 75% onset asynchrony, *p* < 0.005) than all other asynchronies. In contrast, when the first response was delayed, recognition was significantly better than all other asynchronies (all *p* < 0.005) when the speech was centered in time with the text presentation (25% onset asynchrony).

### 3.2 Comparison to unimodal performance

As the multimodal stimulus provides both speech and text, multimodal performance was compared to unimodal speech and text recognition to examine the multimodal benefit. In comparison to unimodal text, recognition of text-supplemented speech was better at the first interval for the delayed condition [*t*(19) = 23.9, *p* < 0.001, *d* = 5.4], but only better at the second interval for the simultaneous condition [*t*(19) = 12.6, *p* < 0.001, *d* = 2.8]. Similarly, in comparison to unimodal speech, text-supplementation resulted in better recognition at the first interval for the delayed condition [*t*(19) = 9.5, *p* < 0.001, *d* = 2.1], but recognition was only better at the second interval for the simultaneous condition [*t*(19) = 9.0, *p* < 0.001, *d* = 2.0], suggesting text supplementation facilitates speech recognition, although additional processing time may be required to obtain the full benefits during simultaneous processing.

### 3.3 Error analysis

**Fig. 3.**
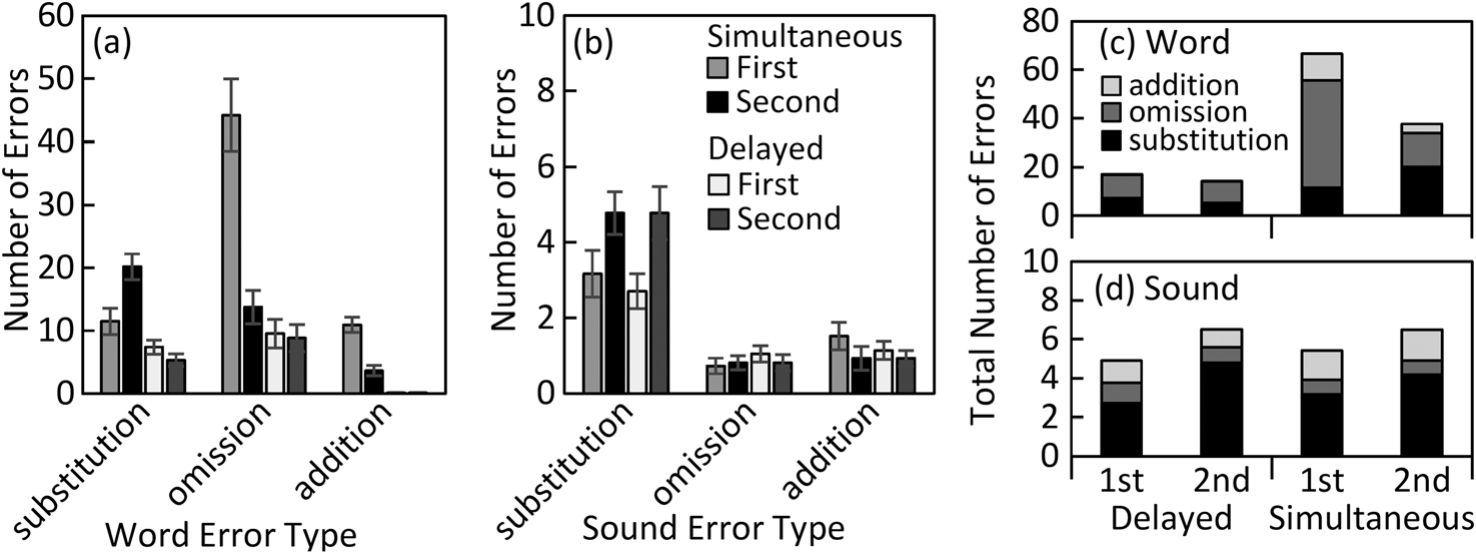
The mean number of (a) word and (b) sound errors in each error type (substitution, omission, addition) for the two response timings (simultaneous, delayed) at the two recall intervals (first, second) collapsed across modality onset asynchrony. Panels (c) and (d) replot the same word (c) and sound (d) errors as a stacked bar representation to visualize the total errors and proportion of error responses within each category. Note the different scale for word and sound errors [(a),(b) and (c),(d)]. Error bars indicate ±1 standard error of the mean.

Figure 3 shows two visualizations of the mean number of word and sound errors in each error type for the two response timings at the two recall intervals collapsed across modality onset asynchrony. Panels (a) and (b) show the errors as a cluster bar representation and panels (c) and (d) as a stacked bar representation to visualize the total errors and proportion of error responses within each category. Here, we focus on the primary observations, while the full analysis and additional plots are available in the supplementary material.^2^ Two observations are important regarding this error analysis. First, for the simultaneous response, the first recall interval was dominated by word omissions. While word omissions were greatly reduced by the second recall interval, word substitutions were produced with greater frequency at the second interval. Omissions [Fig. 3(c), gray bars] account for the greatest proportion of total word errors (66%) at the first interval [see also Fig. 3(a), left most bar for each error type], while substitutions [Fig. 3(c), black bars] accounted for the greatest proportion of word errors at the second interval (54%, from 17% in the first interval). This was seen as a significant interaction in the repeated-measures ANOVA between interval and error type [*F*(2,38) = 38.1, *p* < 0.001, η_p_^2^ = 0.67]. Furthermore, the reduction in word omissions was significantly correlated with an increase in word substitutions (Fig. S1; *r* = −0.46, *p* = 0.04). Second, for both response times, there was a decrease in total word errors for the second interval [delayed: *t*(19) = 8.6, *p* < 0.001, *d* = 1.9; simultaneous: *t*(19) = 6.2, *p* < 0.001, *d* = 1.4]. However, that decrease in total word errors was accompanied by an increase in sound substitutions [delayed: *t*(19) = 7.7, *p* < 0.001, *d* = 1.7; simultaneous: *t*(19) = 2.0, *p* = 0.056, *d* = 0.5]. Importantly, the reduction in total word errors was correlated with the increase in sound substitutions (Fig. S2; delayed: *r* = −0.79, *p* < 0.001; simultaneous: *r* = –0.57, *p* = 0.009). Significant correlations were only observed within task, indicating a shift in error type within the task.

## 4. Discussion

Four primary findings from this study, particularly informed by the error analysis, demonstrate that listeners actively revise their initial interpretations of the sentence when provided additional processing time. First, online shadowing of the stimulus during the simultaneous response resulted in higher errors, mainly word omissions, that were corrected when the response interval was delayed following the stimulus. Second, sentence recognition improved with a reduction in errors from the first to the second recall interval, particularly when the first response was spoken simultaneously with the speech stimulus. Third, in the simultaneous condition, by the time of the second response, listeners corrected initial word omission errors made during the first interval or formed a hypothesis of the missing word through a word substitution. Fourth, across conditions listeners demonstrated revision of initial word errors or made closer approximations with sound errors that were closer to the phonological neighborhood of the target than the initial word error.

Overall, these findings demonstrate that sentence recognition is flexible. When provided additional processing time with the second response interval listeners were able to access initial interpretations in working memory and revise them to reduce errors and respond with closer approximations of the target sentence. This offline revision process of manipulating items in working memory was observable through the use of multiple response intervals and the error analysis. Importantly, the error corrections observed were internal to the listener; sentence presentations were not repeated, and listeners were not given feedback as to the correctness of the response.

These findings replicate and extend our earlier findings (Smith and Fogerty, 2015, 2016). Furthermore, this study is consistent with results suggesting the importance of working memory abilities in the recognition of degraded speech [e.g., Gordon-Salant and Cole (2016); although see Füllgrabe and Rosen (2016)] and speech recognition models that suggest a role of working memory in matching the heard speech to internal lexical representations [e.g., ELU; Rönnberg *et al.* (2013)]. Importantly, increased cognitive load in working memory, associated with greater listening effort [e.g., FUEL; Pichora-Fuller *et al.* (2016)] required to repair incoherent sentence interpretations (Winn and Teece, 2021), results in greater processing time for word recognition (Hadar *et al.*, 2016). By providing additional processing time in this study through delayed and second recall intervals, listeners were able to repair an initially incorrect sentence interpretation with errors shifting from predominately word omissions to sound substitutions, a response closer to the target. This is consistent with the use of top-down lexical information in recovering from the perceptual uncertainty of the degraded words (McClelland and Elman, 1986). Further work will need to investigate if and how this repair process may be affected for individuals with varying sensory or cognitive ability, which may be particularly important, as neurotypical older adults have age-related slowing and reductions in the cognitive resources (e.g., speed of processing, working memory), required for processing natural real-time speech (Salthouse, 1991; Wingfield *et al.*, 2005).

Regarding the multimodal nature of the stimuli, this study examined the relative timing of the two modalities. Consistent with Zekveld and colleagues (2008), minimizing modality asynchrony is essential. For simultaneous responses, recognition was best for synchronous onsets. For delayed responses, a short text lead (at 25% onset asynchrony relative to the text duration), was most beneficial. Of course, this leading text presentation could only occur if the message was known prior to being spoken, such as when the spoken speech is scripted (in which case the text could also be error free, minus any deviations the talker might make while speaking). However, regardless of the modality timing, delaying the response always yielded higher recognition. An important consideration in the current study is that the degradation of the speech and text signals was uncorrelated; however, in practice, masked intervals in the speech would result in corresponding errors in the speech-to-text recognition. Further work will need to investigate how recognition errors may be affected when the degradation between modalities is correlated. The interruption paradigm models the partial information available due to incomplete or partially incorrect text transcription, or partial processing due to sensory or cognitive limitations. Future work will also need to extend these results to more real-world speech and text degradations, especially where transcription errors might provide conflicting cues.

Overall, the results from this study are consistent with the use of cognitive repair in the recognition of interrupted speech and text [e.g., Besser *et al.* (2013)]. When provided additional processing time, listeners are able to correct initial sentence interpretations in working memory, resulting in sentence recognition that improved in word accuracy and which had errors that were closer approximations to the sound structure of the target words. The current results document the importance of processing time and working memory resources in the recognition of text-supplemented speech.

## Footnotes

^1^
The text was visually available for only part of the speech duration in the 75% onset asynchrony condition due to the partial overlap of modalities.

^2^
See supplementary material at https://www.scitation.org/doi/suppl/10.1121/10.0017215 for the complete results of the error analysis and corresponding correlation plots.
